# Delayed Spine Pruning of Direct Pathway Spiny Projection Neurons in a Mouse Model of Parkinson’s Disease

**DOI:** 10.3389/fncel.2019.00032

**Published:** 2019-02-12

**Authors:** Steven M. Graves, D. James Surmeier

**Affiliations:** ^1^Department of Pharmacology, University of Minnesota, Minneapolis, MN, United States; ^2^Department of Physiology, Feinberg School of Medicine, Northwestern University, Chicago, IL, United States

**Keywords:** striatum, spiny projection neurons, Parkinson’s disease, spine density, excitability

## Abstract

In animal models of Parkinson’s disease (PD), principal striatal spiny projection neurons (SPNs) lose axospinous synapses. However, there has been a disagreement about whether this loss is restricted to a specific type of SPN or not, as some studies have reported pruning in both direct pathway SPNs and indirect pathway SPNs, while others have found this pruning to be restricted to indirect pathway SPNs. One possible explanation for the discrepancy is the period between the induction of the parkinsonian state and the assessment of spine loss. To test this hypothesis, transgenic mice were subjected to unilateral 6-hydroxydopamine (6-OHDA) lesions of nigrostriatal dopaminergic neurons and then direct pathway SPNs examined in *ex vivo* brain slices using two photon laser scanning microscopy either one or 2 months afterwards. These studies revealed that 1 month after the lesion, there was no loss of spines in direct pathway SPNs. However, 2 months after the lesion, spine loss was significant in direct pathway SPNs. In addition to reconciling the existing literature on the impact of the parkinsonian state on axospinous synapse elimination in SPNs, our results suggest that the delayed spine loss in direct pathway SPNs is not driven by homeostatic mechanisms [as posited for indirect pathway (iSPNs)], but rather by network pathophysiology.

## Introduction

The striatum is the largest of the basal ganglia nuclei that control goal-directed movement and habit. The principal neurons of the striatum are GABAergic spiny projection neurons (SPNs). Approximately half of SPNs express D1 dopamine receptors (D1Rs) and project to the substantia nigra pars reticulata forming the direct pathway (dSPNs), whereas the remaining SPNs express D2 dopamine receptors (D2Rs) and project to the globus pallidus external segment forming the indirect pathway (iSPNs; Gerfen and Surmeier, 2011). Both SPN populations have dendritic arbors that are densely populated with spines forming cortical and thalamic glutamatergic synapses (Deng et al., 2013; Fieblinger et al., 2014).

In Parkinson’s disease (PD), the dopaminergic innervation of the striatum is lost resulting in a range of adaptations (Zhai et al., 2018). One adaptation that is common to the striatum of PD patients (McNeill et al., 1988; Stephens et al., 2005; Zaja-Milatovic et al., 2005) as well as non-human primate (Villalba et al., 2009) and rodent PD models (Ingham et al., 1989; Day et al., 2006; Zhang et al., 2013; Fieblinger et al., 2014; Suarez et al., 2014, 2016, 2018; Toy et al., 2014; Ueno et al., 2014) is the loss of SPN axospinous synapses. However, it remains unclear whether spine pruning occurs in both SPN populations. Several studies have argued that spine pruning occurs exclusively in iSPNs (Day et al., 2006; Fieblinger et al., 2014; Ueno et al., 2014), whereas other studies suggest that pruning occurs in iSPNs and dSPNs (Villalba et al., 2009; Suarez et al., 2014, 2016, 2018; Toy et al., 2014; Gagnon et al., 2017).

One variable that might account for the discrepancy is the length of time between the lesion and the assessment of spine loss. In most of the studies reporting selective pruning, this period was 1 month; but in many of the studies finding less selectivity, this period was considerably longer. The present study was designed to test this hypothesis by assaying dSPN excitability and dendritic spine density 30 and 60 days after a 6-hydroxydopamine (6-OHDA) lesion. Our results show that dSPN spines are pruned but that this occurs much more slowly than that reported in iSPNs (Fieblinger et al., 2014). This difference in time-course suggests that the mechanisms driving dSPN spine loss differ from those in iSPNs.

## Methods

### Animals

Male mice (*n* = 27) with the TdTomato fluorescent reporter encoded under the *drd1a* receptor regulatory element to identify dSPNs were bred in-house; experimental subjects were hemizygous. Procedures were approved by the Northwestern University Animal Care and Use Committees and in accordance with the National Institutes of Health Guide for the Care and Use of Laboratory Animals. Animals were group housed on a 12-h light/dark cycle with free access to food and water. Mice were 6–10 weeks of age at the time of 6-OHDA lesion.

### Unilateral 6-OHDA Lesion

Mice were anesthetized using an isoflurane vaporizer (Kent Scientific) and placed in a stereotaxic frame (David Kopf Instruments) with a Cunningham adaptor (Harvard Apparatus) while a small incision was made and a hole drilled overlying the medial forebrain bundle. A calibrated glass micropipette (Drummond Scientific Company) pulled on a P-97 micropipette puller (Sutter Instruments) was filled with 0.02% ascorbic acid in sterile saline (vehicle) or 6-OHDA (3.5 μg in vehicle) was lowered into the medial forebrain bundle (AP: −0.7, ML: 1.2, DV: −4.75) and 1 μl injected. After stereotaxic injection mice were monitored and provided saline injections and/or high-fat/high-sucrose food on an as needed basis.

### *Ex vivo* Slices

Mice were anesthetized 30 or 60 days post stereotaxic injection with ketamine (100 mg/kg)/xylazine (7 mg/kg) and transcardially perfused with ice cold modified artificial cerebrospinal fluid (aCSF) containing in mM: 124.0 NaCl, 3.0 KCl, 1.0 CaCl_2_, 2.0 MgCl_2_, 26 NaHCO_3_, 1.0 NaH_2_PO_4_, and 16.66 glucose. Saggital slices from the lesioned hemisphere containing the dorsolateral striatum (275 μm thick) were sectioned using a vibratome (Leica Biosystems) and transferred to a holding chamber (34 degrees Celsius) with aCSF containing in mM: 124.0 NaCl, 3.0 KCl, 2.0 CaCl_2_, 1.0 MgCl_2_, 26 NaHCO_3_, 1.0 NaH_2_PO_4_, and 16.66 glucose for 30–40 min prior to experimentation; solutions were pH 7.4, 310–320 mOsm and continually bubbled with 95% O_2_/5% CO_2_.

### Two-Photon Laser Scanning Microscopy (2PLSM) and Electrophysiology

Slices were placed in a recording chamber and dSPNs visualized by somatic tdTomato with a laser scanning microscope system (Bruker) using 810 nm excitation by a two-photon laser (Coherent Inc.). Identified dSPNs were patched under an Olympus 60×/0.9 NA lens using glass pipettes (3–4.5 MΩ resistance) filled with recording solution containing (in mM): 135.0 KMeSO4, 5.0 KCL, 10.0 HEPES, 2.0 Mg-ATP, 0.5 Na-GTP, 5 phosphocreatine-Tris, 5.0 phosphocreatine-Na, 0.1 spermine; pH was adjusted to 7.25–7.30 and osmolarity 270–280 mOsm. Alexa 568 (50 μM) and Fluo-4 (200 μM) dyes were included in recording pipettes. Whole-cell patch clamp recordings were obtained at 32–34 degrees Celsius using a Multiclamp 700B amplifier as previously described (Fieblinger et al., 2014). Intrinsic excitability was assessed in current clamp with 500 ms somatic current injections (25 pA steps). Dendritic excitability was determined by somatic current injection evoking back propagating action potentials (bAPs) and measuring Fluo-4 fluorescence signals in proximal (30–60 μm from the soma) and distal (>80 μm from the soma) dendrites and spines using 2PLSM (Day et al., 2008). Data are presented as the area of the fluorescence change in distal divided by the proximal fluorescence change; this ratio was called the dendritic index and was used to correct for differences in dye loading, laser power and optical path. After electrophysiological recordings, *z*-series were obtained of proximal and distal dendritic segments as previously described (Fieblinger et al., 2014). High magnification images were acquired with 0.15 μm × 0.15 μm resolution at 0.3 μm *z*-steps; images were processed using AutoQuant deconvolution software (Media Cybernetics) and semi-automated spine counting using NeuronStudio (CNIC). Whole cell *z*-series were acquired at 0.389 μm × 0.389 μm resolution with 0.5 μm steps for Sholl’s analysis using Neurolucida (MBF Bioscience).

### Statistical Analysis

Non-parametric statistics were used for displaying data and for hypothesis testing with GraphPad Prism (GraphPad Software). Kruskal-Wallis with Dunn’s *post hoc* analysis was performed with data expressed as median, quartiles, and range unless otherwise stated; *α* = 0.05.

## Results

### Physiological Adaptations in dSPNs From Parkinsonian Mice

**Figure 1:**
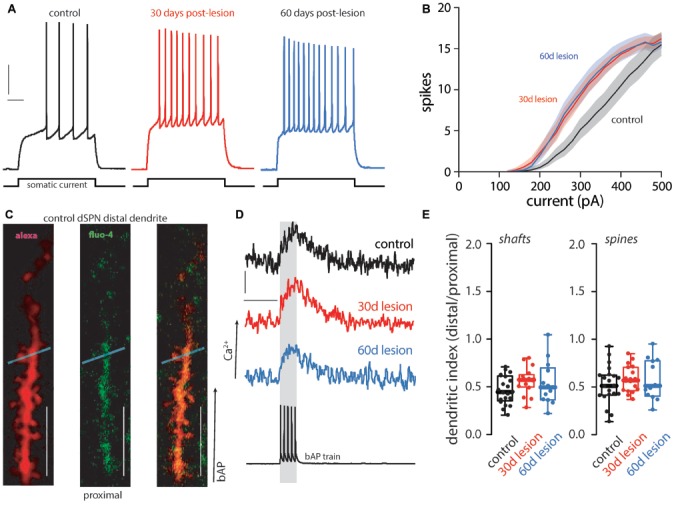
Functional changes of direct pathway spiny projection neurons (dSPNs) in the 6-hydroxydopamine (6-OHDA) mouse model of Parkinson’s disease (PD). Intrinsic and dendritic excitability was assessed in dSPNs in *ex vivo* slices from control mice and compared to slices prepared from mice 30 (30 day lesion) or 60 (60 days lesion) days post 6-OHDA lesion of the medial forebrain bundle. Intrinsic excitability was assessed by somatic current injection. **(A)** dSPN intrinsic excitability was increased in brain slices from parkinsonian mice at 30 and 60 days post-lesion. Sample traces at 300 pA current injection illustrating changes in excitability are provided with vertical and horizontal scale bars indicating 10 mV and 100 ms, respectively. **(B)** Current response curves (shaded regions depict SEM). Somatic excitability was increased in dSPNs from lesioned mice at 30 and 60 days post-lesion compared to controls (control *n* = 21 neurons, 30 days lesion *n* = 19 neurons, and 60 days lesion *n* = 10 neurons). **(C)** Dendritic excitability was determined by current injection and measurement of back propagating calcium signals using a fluorescent dye (fluo-4) at distal and proximal dendrites. Sample two-photon images are provided of a distal dendritic segment filled with an anatomical dye (alexa 568; left), calcium sensitive dye (fluo-4; middle), and the merged image (right). The light blue line depicts the region scanned to acquire fluorescent measurements in the dendritic shaft and spine; scale bar denotes 10 μm. **(D)** Sample traces illustrating the somatic voltage recording (bottom) and the corresponding calcium signal in a distal dendrite in dSPNs from control, 30 days lesion, and 60 days lesion subjects; scale bars denote 200 fluorescent units and 200 ms, respectively. **(E)** Data is presented as the polarization index (i.e., distal/proximal measurements; see methods). There was no difference in dendritic excitability in the shaft or spine (control *n* = 21 neurons, 30 days lesion *n* = 17 neurons, and 60 days lesion *n* = 13 neurons).

Previously it was reported that the intrinsic excitability of dSPNs was elevated 1 month after a unilateral 6-OHDA lesion, but dendritic excitability was unchanged (Fieblinger et al., 2014; Suarez et al., 2016, 2018). This finding was reproduced here. Thirty days after lesioning, the intrinsic excitability of dSPNs was increased (Figures 1A,B) and persisted 2 months post-lesion (Figures 1A,B). In contrast, dendritic excitability as assessed by ratio of the distal Ca^2+^ transient evoked by back-propagating action potentials to that in the proximal dendrites (ratio = dendritic index), was unchanged, regardless of whether the measurement was made in dendritic shafts or spines (Figures 1C–E). Two months following the lesion, dendritic excitability remained similar to that of dSPNs from unlesioned mice (Figures 1C–E).

### Dendritic Adaptations in dSPNs From Parkinsonian Mice

**Figure 2:**
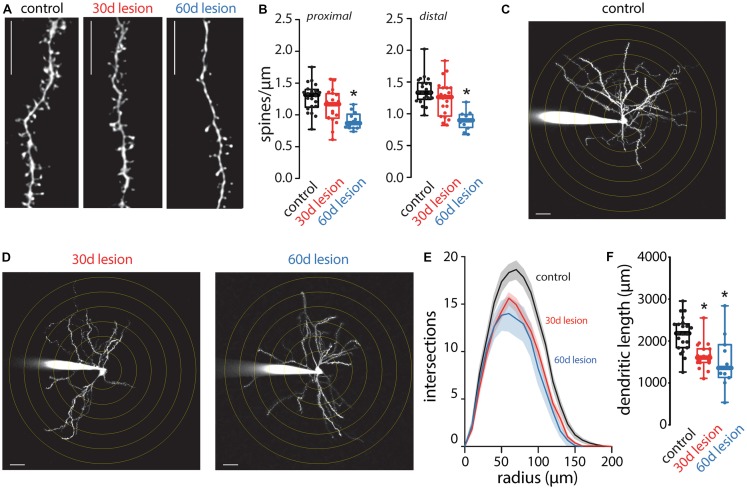
Anatomical changes of dSPNs in the 6-OHDA lesion mouse model of PD. Spine density and dendritic arborization were measured in dSPNs in *ex vivo* slices obtained from control mice and compared to slices prepared from mice 30 (30 days lesion) or 60 (60 days lesion) days post 6-OHDA lesion of the medial forebrain bundle. **(A)** Sample two-photon images of distal dendritic segments illustrating reduced spine density at 60 days lesion compared to control but no change at 30 days lesion; scale bar denotes 10 μm. **(B)** Quantified spine densities from proximal and distal dendritic segments. dSPN spine density was decreased at 60 days post-lesion compared to control with no change at 30 days post lesion (proximal: control *n* = 22 neurons, 30 days lesion *n* = 20 neurons, and 60 days lesion *n* = 13 neurons; distal: control *n* = 21 neurons, 30 days lesion *n* = 20 neurons, and 60 days lesion *n* = 12 neurons). **(C)** Sample two-photon image of a dSPN from a control mouse. Concentric circles are spaced 10 μm apart and scale bar denotes 10 μm. **(D)** Sample two-photon images of dSPNs at 30 and 60 days post-lesion; dendritic arborization is reduced at both time points compared to control image presented in **(C)**. **(E)** Sholl analysis of dSPNs from control (*n* = 22 neurons), 30 days lesion (*n* = 19 neurons) and 60 days lesion (*n* = 11 neurons); solid lines represent the mean and shaded lines the SEM. **(F)** Total dendritic length was reduced in slices obtained from 30 days lesion and 60 days lesion mice compared to controls (control *n* = 22 neurons, 30 days lesion *n* = 19 neurons, and 60 days lesion *n* = 11 neurons); *p < 0.05.

Following short-term (5-day) dopamine depletion using reserpine or 1 month after a unilateral 6-OHDA lesion, spine loss is restricted to iSPNs (Day et al., 2006; Fieblinger et al., 2014). In contrast, dSPN spine pruning has been seen in 1-methyl-4-phenyl-1,2,3,6-tetrahydropyridine (MPTP)-lesioned non-human primates and MPTP or 6-OHDA lesioned mice when examined at longer times after the insult (Villalba et al., 2009; Suarez et al., 2014, 2016; Toy et al., 2014; Gagnon et al., 2017). Here, there was no change in dSPN spine density when measured 1 month after the 6-OHDA lesion (Figures 2A,B). However, when spine density was measured 2 months after the lesion, the loss of dSPN spines was evident in both proximal and distal dendrites (Figures 2A,B). Approximately one third of dSPN spines were lost 2 months after lesioning, an effect size similar to that previously reported for iSPNs (Fieblinger et al., 2014).

In contrast to the slowly developing pruning of dSPN spines, dSPN dendritic atrophy manifested rapidly. One month post-lesion, dendrites of dSPNs were reduced in complexity (Figures 2C–F), as reported previously (Fieblinger et al., 2014). It is important to note that although spine density was not changed 1 month post-lesion, the total number of spines would be reduced as a result of decreased total dendritic length as previously reported (Fieblinger et al., 2014). The dendritic atrophy was evident in both the Sholl plots, which measure branching, and in estimates of total dendritic length. Two months after lesioning, the deficit in dendritic branching and length was similar to that seen 1 month after lesioning (Figures 2C–F).

## Discussion

Our main finding is that dSPNs do lose spines in a mouse model of PD. However, this loss lags behind that reported for iSPNs (Fieblinger et al., 2014). It also lags behind changes in the physiology and global dendritic architecture of dSPNs. While our results argue that methodological differences, particularly the time post-lesion in which experiments are conducted, are responsible for the apparent discrepancy in the literature on PD-related spine loss in iSPNs and dSPNs, they also suggest that the biological determinants of spine loss in these two classes of striatal neurons are quite different with dSPN changes being partially homeostatic.

When the spike rate of neurons is pushed away from a phenotypically determined set point, homeostatic mechanisms are engaged to normalize the spike rate (Marder and Goaillard, 2006; Turrigiano, 2008). These homeostatic mechanisms are capable of up-regulating or down-regulating synaptic connections, as well as changing intrinsic ionic conductances that govern spiking. Dopamine depletion deprives dSPNs of D1R activation and intracellular signaling that serves to increase intrinsic excitability and promote long-term potentiation (LTP) of axospinous glutamatergic synapses (Shen et al., 2008, 2015) resulting in a “dis-facilitation” that would reduce spike rate. Consistent with this there is a reduction in dSPN corticostriatal responses in lesioned rodents (Flores-Barrera et al., 2010; Escande et al., 2016). The up-regulation in intrinsic excitability of dSPNs in the following dopamine depletion (Fieblinger et al., 2014; Suarez et al., 2016) can thus be viewed as a homeostatic adaptation. What is lacking from a homeostatic response is an up-regulation of spine number. Why this is the case is not clear. It is possible that a homeostatic up-regulation in the number of glutamatergic synapses is counter-balanced by the loss of axospinous synapses that depend upon D1R signaling for maintenance (Plotkin et al., 2014). If this is the case, the afferent connectome of dSPNs would be expected to change in the parkinsonian state.

What accounts for the delayed loss of dSPN spines? In the parkinsonian striatum, the loss of D2R signaling dis-inhibits iSPNs and cholinergic interneurons that modulate both iSPN and dSPN function. In iSPNs cholinergic disinhibition increases dendritic excitability and spine pruning *via* M1 receptors (Shen et al., 2007). In dSPNs, M4 muscarinic receptors (M4Rs) oppose D1R signaling and promote cannabinoid-dependent LTD at axospinous synapses (Shen et al., 2015). Further study is needed to determine whether M4R signaling contributes to the loss of axospinous synapses in dSPNs. Moreover, it will be important to determine the functional properties of the synapses that are lost. In naïve dSPNs, approximately one-third of the axospinous synapses appear capable of undergoing D1R-dependent LTP (Plotkin et al., 2014)–precisely the percentage of axospinous synapses pruned by prolonged dopamine depletion. Investigation is needed to determine whether the spines lost are LTP capable or not in order to more fully understand the consequence of dopamine depletion on basal ganglia function and motor dysfunction.

Overall the loss of axospinous synapses in dSPNs would serve to exacerbate pathway imbalance underlying parkinsonian motor symptoms. The loss of D2R activation in iSPNs dis-inhibits them by shutting down intracellular signaling cascades that dampen intrinsic excitability and stimulate the production of endocannabinoids that diminish glutamate release and promote long-term depression (LTD) of axospinous glutamatergic synapses (Wang et al., 2006; Kreitzer and Malenka, 2007; Shen et al., 2008; Thiele et al., 2014). This dis-inhibition of the indirect pathway is thought to contribute to the hypokinetic symptoms of dopamine depletion. The “dis-facilitation” of dSPNs complements the dis-inhibition in iSPNs to promote the indirect pathway dominance thought to underlie bradykinesia and rigidity characteristic of PD.

## Data Availability

Data presented in the manuscript are available from the corresponding author upon request.

## Author Contributions

SG performed experiments and data analysis. SG and DS designed the experiments, drafted and edited the manuscript. The final manuscript was read by and approved by both the authors.

## Conflict of Interest Statement

The authors declare that the research was conducted in the absence of any commercial or financial relationships that could be construed as a potential conflict of interest.

## References

[B1] Day M. Wang Z. Ding J. An X. Ingham C. A. Shering A. F.. (2006). Selective elimination of glutamatergic synapses on striatopallidal neurons in Parkinson disease models. Nat. Neurosci. 9, 251–259. 10.1038/nn1632 16415865

[B2] Day M. Wokosin D. Plotkin J. L. Tian X. Surmeier D. J. (2008). Differential excitability and modulation of striatal medium spiny neuron dendrites. J. Neurosci. 28, 11603–11614. 10.1523/jneurosci.1840-08.2008 18987196 PMC3235729

[B3] Deng Y. P. Wong T. Bricker-Anthony C. Deng B. Reiner A. (2013). Loss of corticostriatal and thalamostriatal synaptic terminals precedes striatal projection neuron pathology in heterozygous Q140 Huntington’s disease mice. Neurobiol. Dis. 60, 89–107. 10.1016/j.nbd.2013.08.009 23969239 PMC3808190

[B4] Escande M. V. Taravini I. R. Zold C. L. Belforte J. E. Murer M. G. (2016). Loss of homeostasis in the direct pathway in a mouse model of asymptomatic Parkinson’s disease. J. Neurosci. 36, 5686–5698. 10.1523/jneurosci.0492-15.2016 27225760 PMC6601837

[B5] Fieblinger T. Graves S. M. Sebel L. E. Alcacer C. Plotkin J. L. Gertler T. S.. (2014). Cell type-specific plasticity of striatal projection neurons in parkinsonism and L-DOPA-induced dyskinesia. Nat. Commun. 5:5316. 10.1038/ncomms6316 25360704 PMC4431763

[B6] Flores-Barrera E. Vizcarra-Chacon B. J. Tapia D. Bargas J. Galarraga E. (2010). Different corticostriatal integration in spiny projection neurons from direct and indirect pathways. Front. Syst. Neurosci. 4:15. 10.3389/fnsys.2010.00015 20589098 PMC2893005

[B7] Gagnon D. Petryszyn S. Sanchez M. G. Bories C. Beaulieu J. M. De Koninck Y.. (2017). Striatal neurons expressing D_1_ and D_2_ receptors are morphologically distinct and differently affected by dopamine denervation in mice. Sci. Rep. 7:41432. 10.1038/srep41432 28128287 PMC5269744

[B8] Gerfen C. R. Surmeier D. J. (2011). Modulation of striatal projection systems by dopamine. Annu. Rev. Neurosci. 34, 441–466. 10.1146/annurev-neuro-061010-113641 21469956 PMC3487690

[B9] Ingham C. A. Hood S. H. Arbuthnott G. W. (1989). Spine density on neostriatal neurones changes with 6-hydroxydopamine lesions and with age. Brain Res. 503, 334–338. 10.1016/0006-8993(89)91686-7 2514009

[B10] Kreitzer A. C. Malenka R. C. (2007). Endocannabinoid-mediated rescue of striatal LTD and motor deficits in Parkinson’s disease models. Nature 445, 643–647. 10.1038/nature05506 17287809

[B11] Marder E. Goaillard J. M. (2006). Variability, compensation and homeostasis in neuron and network function. Nat. Rev. Neurosci. 7, 563–574. 10.1038/nrn1949 16791145

[B12] McNeill T. H. Brown S. A. Rafols J. A. Shoulson I. (1988). Atrophy of medium spiny I striatal dendrites in advanced Parkinson’s disease. Brain Res. 455, 148–152. 10.1016/0006-8993(88)90124-2 3416180

[B13] Plotkin J. L. Day M. Peterson J. D. Xie Z. Kress G. J. Rafalovich I.. (2014). Impaired TrkB receptor signaling underlies corticostriatal dysfunction in Huntington’s disease. Neuron 83, 178–188. 10.1016/j.neuron.2014.05.032 24991961 PMC4131293

[B14] Shen W. Flajolet M. Greengard P. Surmeier D. J. (2008). Dichotomous dopaminergic control of striatal synaptic plasticity. Science 321, 848–851. 10.1126/science.1160575 18687967 PMC2833421

[B15] Shen W. Plotkin J. L. Francardo V. Ko W. K. Xie Z. Li Q.. (2015). M4 muscarinic receptor signaling ameliorates striatal plasticity deficits in models of L-DOPA-induced dyskinesia. Neuron 88, 762–773. 10.1016/j.neuron.2015.10.039 26590347 PMC4864040

[B16] Shen W. Tian X. Day M. Ulrich S. Tkatch T. Nathanson N. M.. (2007). Cholinergic modulation of Kir2 channels selectively elevates dendritic excitability in striatopallidal neurons. Nat. Neurosci. 10, 1458–1466. 10.1038/nn1972 17906621

[B17] Stephens B. Mueller A. J. Shering A. F. Hood S. H. Taggart P. Arbuthnott G. W.. (2005). Evidence of a breakdown of corticostriatal connections in Parkinson’s disease. Neuroscience 132, 741–754. 10.1016/j.neuroscience.2005.01.007 15837135

[B18] Suarez L. M. Alberquilla S. Garcia-Montes J. R. Moratalla R. (2018). Differential synaptic remodeling by dopamine in direct and indirect striatal projection neurons in Pitx3(−/−) mice, a genetic model of Parkinson’s disease. J. Neurosci. 38, 3619–3630. 10.1523/JNEUROSCI.3184-17.2018 29483281 PMC6705913

[B19] Suarez L. M. Solis O. Aguado C. Lujan R. Moratalla R. (2016). L-DOPA oppositely regulates synaptic strength and spine morphology in D1 and D2 striatal projection neurons in dyskinesia. Cereb. Cortex 26, 4253–4264. 10.1093/cercor/bhw263 27613437 PMC5066835

[B20] Suarez L. M. Solis O. Carames J. M. Taravini I. R. Solis J. M. Murer M. G.. (2014). L-DOPA treatment selectively restores spine density in dopamine receptor D2-expressing projection neurons in dyskinetic mice. Biol. Psychiatry 75, 711–722. 10.1016/j.biopsych.2013.05.006 23769604

[B21] Thiele S. L. Chen B. Lo C. Gertler T. S. Warre R. Surmeier J. D.. (2014). Selective loss of bi-directional synaptic plasticity in the direct and indirect striatal output pathways accompanies generation of parkinsonism and l-DOPA induced dyskinesia in mouse models. Neurobiol. Dis. 71, 334–344. 10.1016/j.nbd.2014.08.006 25171793 PMC4486078

[B22] Toy W. A. Petzinger G. M. Leyshon B. J. Akopian G. K. Walsh J. P. Hoffman M. V.. (2014). Treadmill exercise reverses dendritic spine loss in direct and indirect striatal medium spiny neurons in the 1-methyl-4-phenyl-1,2,3,6-tetrahydropyridine (MPTP) mouse model of Parkinson’s disease. Neurobiol. Dis. 63, 201–209. 10.1016/j.nbd.2013.11.017 24316165 PMC3940446

[B23] Turrigiano G. G. (2008). The self-tuning neuron: synaptic scaling of excitatory synapses. Cell 135, 422–435. 10.1016/j.cell.2008.10.008 18984155 PMC2834419

[B24] Ueno T. Yamada J. Nishijima H. Arai A. Migita K. Baba M.. (2014). Morphological and electrophysiological changes in intratelencephalic-type pyramidal neurons in the motor cortex of a rat model of levodopa-induced dyskinesia. Neurobiol. Dis. 64, 142–149. 10.1016/j.nbd.2013.12.014 24398173

[B25] Villalba R. M. Lee H. Smith Y. (2009). Dopaminergic denervation and spine loss in the striatum of MPTP-treated monkeys. Exp. Neurol. 215, 220–227. 10.1016/j.expneurol.2008.09.025 18977221 PMC2680135

[B26] Wang Z. Kai L. Day M. Ronesi J. Yin H. H. Ding J.. (2006). Dopaminergic control of corticostriatal long-term synaptic depression in medium spiny neurons is mediated by cholinergic interneurons. Neuron 50, 443–452. 10.1016/j.neuron.2006.04.010 16675398

[B27] Zaja-Milatovic S. Milatovic D. Schantz A. M. Zhang J. Montine K. S. Samii A.. (2005). Dendritic degeneration in neostriatal medium spiny neurons in Parkinson disease. Neurology 64, 545–547. 10.1212/01.wnl.0000150591.33787.a4 15699393

[B28] Zhai S. Tanimura A. Graves S. M. Shen W. Surmeier D. J. (2018). Striatal synapses, circuits, and Parkinson’s disease. Curr. Opin. Neurobiol. 48, 9–16. 10.1016/j.conb.2017.08.004 28843800 PMC6022405

[B29] Zhang Y. Meredith G. E. Mendoza-Elias N. Rademacher D. J. Tseng K. Y. Steece-Collier K. (2013). Aberrant restoration of spines and their synapses in L-DOPA-induced dyskinesia: involvement of corticostriatal but not thalamostriatal synapses. J. Neurosci. 33, 11655–11667. 10.1523/jneurosci.0288-13.2013 23843533 PMC3724545

